# Ultra-low values of total serum immunoglobulin E and cancer risk: a systematic review and exploratory analysis of the Swedish AMORIS cohort

**DOI:** 10.3389/fimmu.2026.1812536

**Published:** 2026-10-22

**Authors:** Maria J. Monroy-Iglesias, Gincy George, Isolt Reardon, Jitesh Chauhan, Heather J. Bax, Alexandra McCraw, Michelle C. Turner, Aurélie Poli, Niklas Hammar, Maria Feychting, Mats Talbäck, Denisa E. Ferastraoaru, Aida Santaolalla, Sophia N. Karagiannis, Mieke Van Hemelrijck

**Affiliations:** 1 Transforming Cancer OUtcomes through Research (TOUR), School of Cancer and Pharmaceutical Sciences, King’s College London, London, United Kingdom; 2 St. John’s Institute of Dermatology, School of Basic and Medical Biosciences and King's Health Partners (KHP) Centre for Translational Medicine, Guy’s Hospital, King’s College London, London, United Kingdom; 3 Barcelona Institute for Global Health (ISGlobal), Barcelona, Spain; 4 Universitat Pompeu Fabra (UPF), Barcelona, Spain; 5 Centro de Investigación Biomédica en Red (CIBER) Epidemiología y Salud Pública (CIBERESP), Madrid, Spain; 6 Neuro-Immunology Group, Department of Cancer Research, Luxembourg Institute of Health, Luxembourg, Luxembourg; 7 Unit of Epidemiology, Institute of Environmental Medicine, Karolinska Institutet, Stockholm, Sweden; 8 Department of Internal Medicine/Allergy and Immunology, Albert Einstein College of Medicine, Montefiore Medical Center, Bronx, NY, United States; 9 Breast Cancer Now Research Unit, School of Cancer and Pharmaceutical Sciences, King’s College London, Innovation Hub, Guy’s Cancer Centre, London, United Kingdom

**Keywords:** AllergoOncology, AMORIS, biomarkers, cancer risk, IgE, IgE deficiency, ultra-low

## Abstract

**Background:**

Immunoglobulin E (IgE) is increasingly recognized as a component of immune surveillance with potential relevance to cancer biology. While elevated IgE has been studied in relation to cancer risk, the implications of ultra-low or deficient total serum IgE remain unclear.

**Methods:**

We conducted a systematic review of observational studies examining ultra-low or deficient total serum IgE and cancer risk, searching PubMed/MEDLINE and Embase through September 2026. We additionally conducted exploratory analyses among 26,676 adults in the Swedish Apolipoprotein-related Mortality RISk (AMORIS) cohort with baseline total serum IgE measurements. Cancer risk was assessed using Cox proportional hazards models adjusted for age, sex, and education. IgE was analyzed using clinically defined categories (<2, 2–10, 10–20, 20–100, 100–<1,000, ≥1,000kU/L).

**Results:**

Seven observational studies met inclusion criteria. Several studies reported higher cancer prevalence or risk among individuals with ultra-low or deficient IgE, although studies were heterogeneous and largely retrospective or unadjusted. In exploratory AMORIS analyses, there was no evidence of an increased overall cancer risk among individuals with IgE deficiency (<2kU/L).

**Conclusion:**

Existing studies suggest a possible association between ultra-low total serum IgE and cancer risk, but current evidence is limited. In exploratory AMORIS cohort analyses, there was no evidence of an increased overall cancer risk among individuals with IgE deficiency. Further prospective studies with repeated IgE measurements and comprehensive covariate data are needed to clarify this understudied association.

## Introduction

Immunoglobulin E (IgE) is widely recognized as a clinical biomarker of allergic disease. Elevated total and allergen-specific IgE levels are routinely used in diagnostic evaluation, with high total IgE concentrations (typically >100 kU/L) indicating underlying atopy. IgE-mediated mechanisms initiate the cascade of allergic inflammation, giving rise to hallmark manifestations such as asthma, rhinitis, eczema, and anaphylaxis (1). Beyond its established role in allergic disorders, recent advances in the emerging field of AllergoOncology have highlighted a potential involvement of IgE—and allergic inflammation more broadly—in anti-tumor immunosurveillance (2). However, the association between IgE, allergies, and cancer risk remains complex, with prior studies reporting both inverse and positive associations depending, on cancer type anatomical site of the disease and study design (3, 4).

Much of the existing evidence is based on self-reported allergy history, which is susceptible to recall bias and does not always reflect underlying immunological mechanisms (5, 6). Several systematic reviews have documented the heterogeneity of these findings. For instance, a 2016 review of 79 studies found that atopy—defined as heightened IgE responses to allergens—was associated with a lower risk of colorectal and pancreatic cancers but a higher risk of lung and prostate cancers (7). More recently, a 2025 systematic review and meta-analysis of 53 studies reported largely inverse associations between allergic conditions and several cancers (8).

Studies that directly measured total serum IgE levels offer a different and perhaps more objective perspective, but have also yielded inconclusive results. A 2010 meta-analysis by Van Hemelrijck et al. found no overall association between total IgE and cancer risk, though inverse associations were observed for glioma and lymphoma (9). Most prior work has focused on elevated IgE or on comparisons across quartiles within the population distribution, where “normal” IgE values generally range from approximately 2–100 kU/L. Within this range, low IgE is sometimes defined as <20 kU/L, very low IgE as <10 kU/L, and ultra-low IgE as <2.0–2.5 kU/L —a category often referred to as “IgE deficiency” in the clinical literature (10–12). Although typically grouped within the broader normal range, an increasing number of recent studies specifically examining ultra-low IgE now suggest that individuals in this category may have a higher risk of malignancy, with similar patterns observed in both pediatric and adult populations (13, 14).

To examine current evidence on this emerging topic, we conducted a systematic review of studies investigating the association between ultra-low serum IgE and cancer risk. We additionally performed an exploratory analysis using data from the large, prospective AMORIS (Apolipoprotein-related MOrtality RISk) cohort to examine whether pre-diagnostic reduced IgE levels—classified into low, very low, and ultra-low categories—are associated with malignancy risk in adults.

## Methods

### Systematic review

#### Literature search strategy

The systematic review was conducted in accordance with the Preferred Reporting Items for Systematic Reviews and Meta-Analyses (PRISMA) guidelines (**Supplementary Table 1**). A comprehensive literature search was performed in PubMed/MEDLINE and Embase to identify studies evaluating the association between serum IgE deficiency and cancer risk. The PubMed search strategy was: (“Immunoglobulin E” OR IgE OR “total IgE” OR “low IgE” OR “very low IgE” OR “ultra-low IgE” OR “IgE deficiency” OR “IgE deficient” OR “hypo-IgE”) AND (“Neoplasms” OR cancer* OR malignan* OR tumor* OR tumour* OR neoplasm*). The Embase search strategy was: ((IgE OR “immunoglobulin E”) NEAR/3 (low OR deficient OR deficiency OR undetectable OR reduced)) AND (cancer* OR malignan* OR tumor* OR tumour* OR neoplasm*). Both searches were restricted to titles and abstracts. No start date restrictions were applied, and the search included all eligible publications available through September 6^th^, 2026. Reference lists of relevant reviews and meta-analyses identified during screening were also examined to ensure completeness. Grey literature sources, dissertations, and trial registries were not systematically searched.

#### Inclusion criteria

Eligibility criteria were defined according to the PICOS framework. The population included human participants with measured total serum IgE; the exposure was ultra-low or deficient total serum IgE (≤3 kU/L); the comparator was individuals without IgE deficiency; the outcome was cancer diagnosis or incidence; and eligible study designs were observational studies evaluating the association between IgE deficiency and malignancy.

We included human studies published in English that evaluated IgE deficiency, defined by ultra-low total serum IgE levels (typically <2.0–3.0 kU/L), in relation to cancer diagnosis or incidence. Only studies that directly measured total serum IgE were eligible.

Studies that examined IgE as a continuous measure, across population quantiles, or focused on elevated IgE or atopic phenotypes were excluded. Studies focusing exclusively on allergen-specific IgE were also excluded, as these involve different assays and cut-off values that are not comparable to total IgE measurements. We further excluded non-human studies, interventional studies, case reports, case series, commentaries, editorials, letters, conference abstracts, supplements, and narrative reviews that did not present original analytic data. Systematic reviews and meta-analyses were not included as primary evidence but were reviewed to identify potentially relevant original studies not captured in the primary search.

#### Data extraction

Titles and abstracts identified through the search were screened for relevance by two reviewers (MJM, GG). Full-text articles were independently assessed for eligibility by both reviewers. Disagreements regarding study eligibility were resolved through discussion, with consultation of an independent reviewer (AS) when consensus could not be reached. Data extraction was performed by MJM and GG, with each reviewer extracting data from approximately half of the included studies using a standardized, predefined extraction form. Studies meeting the inclusion criteria were retained for qualitative synthesis. From each included study, we extracted information on study author, publication year, study design, population characteristics, sample size, exposure definition and IgE cut-off values, outcome definition, covariates, and key findings. Study quality was assessed using an adapted Newcastle–Ottawa Scale (NOS). Given the heterogeneity of the observational study designs included in the review, the NOS was adapted to allow consistent assessment across studies while retaining its core domains of selection, comparability, and outcome/temporality.

### AMORIS cohort

#### Study population

The study population was drawn from the AMORIS cohort, which includes more than 800,000 Swedish men and women whose blood or urine samples were analyzed at the CALAB clinical laboratory in Stockholm between 1985 and 1996 (9). These samples were obtained as part of routine health examinations in occupational and primary care settings. Through linkage to multiple national registers, the AMORIS cohort has become a valuable resource for studying circulating biomarkers in relation to a wide range of health outcomes, including cancer. Using the unique Swedish 10-digit personal identification number, the cohort was linked to the Swedish National Cancer Registry, the National Cause of Death Register, the National Patient Register, and the National Register of Emigration, with follow-up available through 2011. In addition, data from consecutive Swedish Censuses (1970–1990) and the longitudinal integration database for health insurance and labor market studies (LISA, 1990–2012) were incorporated. A more detailed description of the AMORIS cohort has been published previously (9, 15, 16).

For the present analysis, eligible individuals were aged 18 years or older at baseline, had no prior cancer diagnosis, and had at least one valid serum IgE measurement available, resulting in a study population of 26,676 individuals. All laboratory analyses were performed at the same clinical laboratory (CALAB, Stockholm) using a consistently implemented methodology. Serum IgE levels were assessed using a two-step sandwich ELISA on a fully automated immunoassay system (ES 700; Boehringer–Mannheim, Germany), with a coefficient of variation <5%. Very low IgE measurements were recorded in the AMORIS data as values of approximately 0 kU/L; however, the exact analytical detection limit or lower reportable concentration of the historical assay was not available. Information on IgE (kU/L), age, and sex was obtained from the AMORIS database. Data on education, cancer diagnoses, and mortality were obtained through linkage to national registers. Incident cancers were ascertained from the Swedish National Cancer Register and classified according to the International Classification of Diseases, 7th, 8th, and 9th Revisions (ICD-7, ICD-8, ICD-9).

#### Data analysis

​​In exploratory analyses, Cox proportional hazards regression models were used to estimate hazard ratios (HRs) and 95% confidence intervals (CIs) for the association between IgE levels and overall cancer risk. Follow-up time was defined from the date of IgE measurement until the first occurrence of cancer diagnosis. Subjects were censored at death, emigration, or the end of follow-up.

IgE levels were analyzed using two categorical definitions. The first used cut-points previously applied in clinical and epidemiologic studies (12, 17) to define ultra-low (<2 kU/L), very low (2–10 kU/L), low (10–20 kU/L), normal (20–100 kU/L), high (100–<1,000 kU/L), and very high (≥1,000 kU/L) IgE levels. These categories were used to evaluate associations at clinically relevant IgE thresholds, including the ultra-low levels that were the primary focus of this study. The second definition was based on the distribution of IgE within the study population, with categories defined as the first quartile (<19 kU/L), the second and third quartiles combined (19–128 kU/L), and the fourth quartile (129–15,000 kU/L), allowing an exploratory evaluation of associations across the population IgE distribution independent of predefined clinical thresholds. The population-based analysis was therefore intended as a complementary exploratory analysis rather than an alternative definition of IgE deficiency.

Models were adjusted for age groups (<30, 30-39, 40-49, 50-59, 60-69, ≥70), sex (male, female), and education level (pre-secondary, secondary, and post-secondary), which was included as an available proxy for socioeconomic position. Participants with missing covariate data were excluded from fully adjusted models. Descriptive characteristics were compared across categories of total serum IgE using analysis of variance (ANOVA) for continuous variables and chi-squared tests for categorical variables. The proportional hazards assumption was assessed using Schoenfeld residuals. All analyses were conducted using SAS version 9.1.3 (SAS Institute, Cary, NC) and Stata version 16 (StataCorp, College Station, TX). The AMORIS study was approved by the Regional Ethical Review Board in Stockholm (2010/1047-31/1, 2018/2401-31), that also waived the requirement for participant consent. The study was conducted in accordance with the Declaration of Helsinki.

## Results

### Systematic review

The literature search identified 3,286 records, including 2,823 from PubMed/MEDLINE and 463 from Embase (**Figure 1**). After removal of 109 duplicate records, 3,177 records underwent title and abstract screening, of which 3,123 were excluded. Fifty-four articles were assessed by full-text review, of which seven studies met the inclusion criteria and were included in the final qualitative synthesis (**Table 1**) (14, 18–23).

**Figure 1:**
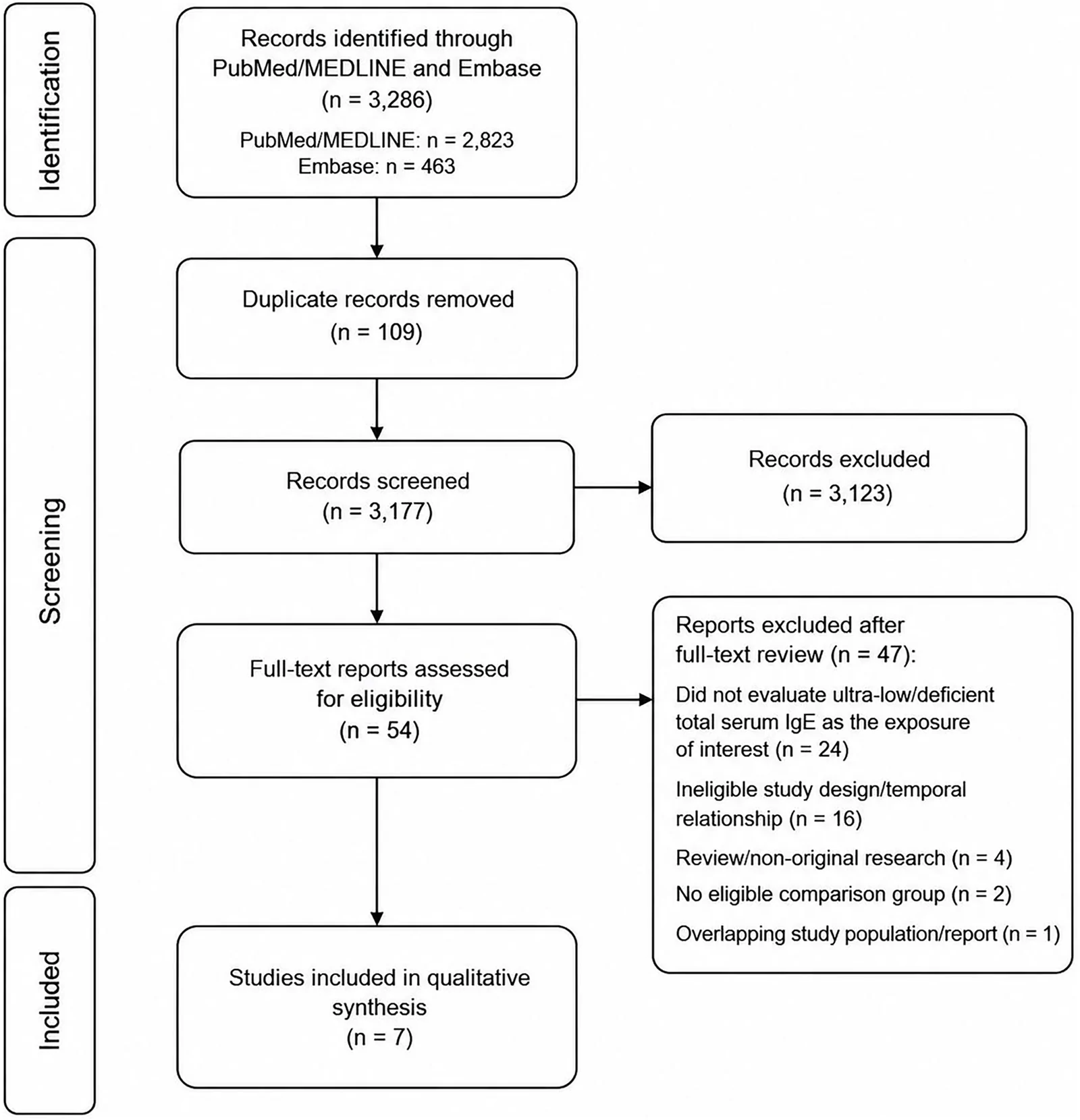
PRISMA flow diagram illustrating study identification, screening, eligibility, and inclusion for the systematic review of ultra-low total serum IgE and cancer risk.

**Table 1:** Summary of cohort characteristics and cancer cases among individuals with ultra-low total serum IgE.

Author, Year	Country	Study type	Population	Total N	IgE-deficient n	IgE cut-off	Cancer cases in IgE-deficient	Follow-up	Outcome	Findings	Effect estimate (95% CI)	Adjustment
Ferastraoaru, 2017 (14)	USA	Retrospective cohort	Adults with total serum IgE	2,339	63	<2.5 kU/L	21	NR	Any cancer	Prior malignancy was more frequent with IgE deficiency (33.3% vs 8.7%).	OR 5.51 (3.07–9.88), IgE-deficient vs non-deficient	Age, sex, smoking
Ferastraoaru, 2018 (18)	USA	Cross-sectional population-based survey (NHANES)	Adults with total serum IgE	4,488	127	<2.5 kU/L	16	N/A (cross-sectional)	Any cancer	Prior malignancy was 12.6% in IgE-deficient participants; comparisons with high and very-high IgE were significant.	OR 1.86 (1.02–3.40) vs high IgE; OR 3.07 (1.03–9.10) vs very high IgE	Age, sex, race, smoking (unweighted)
Ferastraoaru, 2021 (19)	USA	Retrospective cohort	Paediatric patients with total serum IgE	4,586	94	<2.5 kU/L	3	NR	Any cancer	Malignancy was more frequent with IgE deficiency (3.2%) than normal (0.5%), high (0.2%), or very high (0.2%) IgE.	OR 7.84 (1.94–31.61) vs normal IgE	Age, sex, race
Al, 2021 (20)	Turkey	Retrospective descriptive cohort	Paediatric and adult patients with IgE <2 IU/mL	130	130	<2 IU/mL	34	NR	Any cancer	Malignancy was present in 26% of patients with very low IgE.	Not reported (no non-deficient comparator)	Descriptive; no comparative adjustment
Weller, 2023 (21)	USA	Retrospective cohort	Adults with total serum IgE	39,965	2,522	<3 kU/L	225	3.6 years	First malignancy	Undetectable IgE was associated with increased hazard and risk of first malignancy.	Adjusted HR 1.28 (1.08–1.51); adjusted RR 1.49 (1.27–1.75)	BMI, smoking, immunodeficiency, age, sex, race, omalizumab use
Uygun, 2024 (22)	Turkey	Retrospective cohort	Paediatric patients with total serum IgE	6,821	599	<2.5 kIU/L	10	NR	Any cancer	Prior malignancy was more frequent with low IgE (1.7% vs 0.1%).	Adjusted OR 15.07 (5.72–39.75), <2.5 vs ≥2.5 kIU/L	Age, sex, allergy diagnosis
Jevtic, 2026 (23)	USA/Canada	Registry-based retrospective cohort	Patients with primary immunodeficiencies (PID) and available serum IgE	1,593	363	≤2 kU/L	19	NR	Any cancer	No significant association in the overall PID cohort; malignancy was more frequent among IgE-deficient patients in the CVID subgroup.	Overall PID: OR 1.46 (0.78–2.74); CVID: OR 2.89 (1.13–7.46)	Age, sex, race

BMI, body mass index; CI, confidence interval; CVID, common variable immunodeficiency; HR, hazard ratio; IgE, immunoglobulin E; NOS, Newcastle–Ottawa Scale; NR, not reported; OR, odds ratio; PID, primary immunodeficiency; RR, risk ratio.

All included studies were observational studies, published between 2017 and 2026. Study designs included retrospective cohort studies, a cross-sectional population-based survey, and a registry-based retrospective study. Four studies were conducted in adult populations, two in pediatric populations, and one included both adult and pediatric patients. Across studies, IgE deficiency was consistently defined using ultra-low total serum IgE levels, although cut-off values ranged from <2.0 to <3.0 IU/L. All studies evaluated any malignancy as the outcome. Five studies were conducted in the United States and two in Turkey.

Most included studies evaluated prior or prevalent malignancy in relation to IgE deficiency, whereas Weller et al. evaluated the risk of incident malignancy during follow-up. Despite heterogeneity in study populations, study designs, and IgE cut-off definitions, the included studies generally reported a higher prevalence of prior or prevalent malignancy, or a higher risk of incident malignancy, among individuals with IgE deficiency compared with those with normal or higher IgE levels, although the strength and comparability of the evidence varied across studies. Most studies reported unadjusted estimates, three studies adjusted for demographic and/or clinical factors, with the extent of adjustment varying across studies.

Ferastraoaru et al. (2017) (14) conducted a retrospective cohort study among adult patients with measured total serum IgE at a tertiary care center in New York (n = 2,339). IgE deficiency was defined as serum IgE <2.5 kU/L and compared with non–IgE-deficient patients (≥2.5 kU/L). A higher prevalence of prior malignancy was observed among patients with IgE deficiency (21/63, 33%) compared with those without IgE deficiency (197/2,276, 8.7%). Among all 21 IgE-deficient patients with malignancy, IgE was measured after the cancer diagnosis. In stratified analyses, the higher prevalence of malignancy among IgE-deficient individuals persisted both in patients with and without common variable immunodeficiency (CVID), with subgroup-adjusted estimates reported for age, sex, and smoking.

Using data from the 2005–2006 National Health and Nutrition Examination Survey (NHANES), Ferastraoaru et al. (2018) (18) conducted a cross-sectional analysis of malignancy prevalence across IgE categories in a population-based sample of adults (n = 4,488). Participants were categorized as IgE-deficient (<2.5 kU/L), normal (2.5–100 kU/L), high (100–1,000 kU/L), or very high (≥1,000 kU/L). The prevalence of prior malignancy was higher among IgE-deficient individuals (12.6%) compared with those with high (6.7%) or very high IgE levels (5.3%). In exploratory logistic regression analyses adjusting for age, sex, race, and smoking, selected pairwise comparisons suggested higher odds of prior malignancy among IgE-deficient individuals compared with those with high and very high IgE levels. Most malignancies occurred before IgE measurement, with a mean interval of 10.3 years between cancer diagnosis and IgE measurement.

In a subsequent retrospective cohort study, Ferastraoaru et al. (2021) (19) evaluated pediatric patients with available total serum IgE measurements at the same center in New York. Children were similarly categorized into IgE-deficient, normal, high, and very high IgE groups. A higher prevalence of malignancy was observed among IgE-deficient children (3/94, 3.2%) compared with the non-deficient cohort overall (13/4,492, 0.3%). When examined by IgE category, malignancy prevalence was 0.5% in the normal IgE group and 0.2% in both the high and very high IgE groups. All malignancies among IgE-deficient children were lymphomas. Overall, 14 of the 16 children with malignancy had IgE measured after the cancer diagnosis, including two of the three IgE-deficient children with malignancy.

Al et al. (2021) (20) conducted a retrospective descriptive cohort study assessing malignancy prevalence among pediatric and adult patients with IgE deficiency (<2.0 IU/mL) treated at a single center in Turkey between 2015 and 2020. Among 130 patients (57 pediatric and 73 adult), malignancy was diagnosed in 34 individuals (26%), including 9 children (15.7%) and 25 adults (34.2%). Although no comparison group with higher IgE levels was included, malignancy was reported in 26% of IgE-deficient patients. IgE measurements were obtained predominantly before diagnosis or during diagnostic evaluation, although post-diagnosis measurements were also included.

Weller et al. (2023) (21) reported findings from a retrospective cohort study of adults with measured serum IgE levels between 1998 and 2020. Patients with prior malignancy or malignancy diagnosed within three months of the first IgE measurement were excluded, and incident first malignancies occurring during subsequent follow-up were evaluated. In adjusted analyses accounting for age, sex, race, body mass index, smoking status, immunodeficiency, and immunomodulatory therapy use, IgE deficiency (<3.0 IU/L) was associated with an increased risk of any malignancy compared with IgE ≥3.0 IU/L (hazard ratio 1.28; 95% CI 1.08–1.51). Elevated risks were also observed for hematologic malignancies and skin cancers.

Uygun et al. (2024) (22) conducted a retrospective cohort study examining malignancy prevalence among 6,821 pediatric patients with available serum IgE measurements at a tertiary care center in Turkey. Among children with IgE <2.5 kU/L (n = 599), 1.7% (n = 10) had a malignancy, compared with 0.11% (n = 7) among those with IgE ≥2.5 kU/L (n = 6,222). Leukemia accounted for the majority (70%) of malignancies in the IgE-deficient group. In all but one patient with malignancy, IgE was measured after the cancer diagnosis.

Most recently, Jevtic et al. (2026) (23) conducted a registry-based retrospective cohort study evaluating IgE deficiency among 1,593 patients with primary immunodeficiencies enrolled in the U.S. Immunodeficiency Network (USIDNET) registry, of whom 363 had IgE deficiency (≤2 kU/L). Nineteen malignancies were reported among patients with IgE deficiency, compared with 48 among those without IgE deficiency. Among patients with CVID, malignancy was more frequent in those with IgE deficiency than in those without IgE deficiency (9.4% vs 3.5%; OR 2.89, 95% CI 1.13–7.46).

### Quality assessment

Study quality was assessed using an adapted NOS for observational studies. Overall, study quality ranged from low to moderate. Most studies scored well on exposure ascertainment, as all included direct measurement of total serum IgE using standardized assays. However, several studies were limited by their retrospective or cross-sectional design, reliance on prior cancer diagnosis, uncertain temporality between IgE measurement and cancer occurrence, and limited adjustment for potential confounders. Only one study reported fully adjusted risk estimates, while the remaining studies primarily presented unadjusted comparisons. The extent of covariate adjustment varied across studies, with most providing limited or no multivariable adjustment. Domain-specific and overall NOS scores, together with study-specific justifications, are presented in **Supplementary Table 2**.

### The AMORIS cohort

A total of 26,676 individuals from the AMORIS cohort were included, among whom 5,588 incident cancers were diagnosed during a median follow-up of 23 years. Baseline characteristics overall and stratified by total serum IgE levels are shown in **Table 2**. The distribution of demographic and clinical characteristics differed across IgE categories (all P<0.001). Individuals with very high IgE levels (≥1,000 kU/L) were younger and more likely to be male compared with those with ultra-low IgE (<2 kU/L). Conversely, the ultra-low, very low, and low IgE groups had higher proportions of female participants (65%, 69%, and 64%, respectively). A history of pulmonary conditions, particularly asthma, was more common among individuals with elevated IgE levels, whereas educational attainment was broadly similar across IgE categories.

**Table 2:** Characteristics of AMORIS participants by total serum IgE level.

Patient characteristics	Ultra-low IgE (<2 kU/L) n=315 n (%)	Very Low (2-<10 kU/L) n=2,958 n (%)	Low (10-<20 kU/L) n=3,773 n (%)	Normal (20-<100 kU/L) n=11,718 n (%)	High (100-<1000 kU/L) n=7,351 n (%)	Very high (>=1000 kU/L) n=561 n (%)	P-value
Age							
Mean (SD)	42 (15)	43 (15)	43 (15)	42 (15)	40 (15)	39 (15)	
Categories							
<30	85 (27)	715 (24)	879 (23)	2,948 (25)	2,226 (30)	189 (34)	<0.001
30-39	69 (22)	621 (21)	848 (22)	2,741 (23)	1,763 (24)	123 (22)	
40-49	68 (21)	669 (23)	806 (21)	2,613 (22)	1,541 (21)	108 (19)	
50-59	53 (17)	529 (18)	742 (20)	2,026 (17)	1,077 (15)	90 (16)	
60-69	24 (8)	239 (8)	290 (8)	814 (8)	471 (6)	25 (4)	
70+	16 (5)	185 (6)	208 (6)	576 (5)	273 (4)	26 (5)	
Sex							
Male	111 (35)	919 (31)	1,371 (36)	4,661 (40)	3,425 (47)	284 (51)	<0.001
Female	204 (65)	2,039 (69)	2,402 (64)	7,057 (60)	3,926 (53)	277 (49)	
Education							
Pre-secondary	86 (27)	697 (24)	921 (24)	2,949 (25)	1,991 (28)	189 (34)	<0.001
Secondary	143 (45)	1,340 (45)	1,695 (45)	5,268 (45)	3,362 (45)	234 (42)	
Post-secondary	84 (27)	882 (30)	1,117 (30)	3,380 (29)	1,917 (26)	130 (23)	
Missing	2 (1)	39 (1)	40 (1)	121 (1)	81 (1)	8 (1)	
History of pulmonary condition							
Asthma	3 (1)	19 (0.7)	25 (0.7)	99 (0.8)	137 (2)	26 (5)	<0.001
Other	0	9 (0.3)	11 (0.3)	30 (0.2)	42 (1)	4 (1)	
None	312 (99)	2,930 (99)	3,737 (99)	11,589 (99)	7,172 (97)	531 (94)	

IgE, Immunoglobulin E; SD, standard deviation.

Results from Cox proportional hazards models are presented in **Table 3**. Using clinically defined IgE cut-offs, there was no evidence of an increased overall cancer risk among individuals with ultra-low (<2 kU/L), very low (2–10 kU/L), or low (10–20 kU/L) IgE levels and overall cancer risk compared with normal IgE levels. Similarly, high and very high IgE levels were not associated with cancer risk.

**Table 3:** Hazard ratios (HRs) and 95% confidence intervals (CIs) for the association between total serum IgE levels and overall cancer risk in the AMORIS cohort.

Serum IgE levels (kU/L)	No cancer n=20,853 (n, %)	Cancer n=5,532 (n, %)	Unadjusted	Adjusted*
			HR (95%CI)	HR (95%CI)
Clinical cut-offs				
Ultra-low (<2)	253 (81)	60 (19)	0.81 (0.63-1.05)	0.81 (0.62-1.04)
Very low (2-9)	2,246 (77)	673 (23)	1.03 (0.94-1.12)	0.95 (0.87-1.03)
Low (10-19)	2,870 (77)	863 (23)	1.03 (0.95-1.11)	0.95 (0.88-1.02)
Normal (20-99)	9,155 (79)	2,442 (21)	1.00 (Ref)	1.00 (Ref)
High (100-<1000)	5,871 (81)	1,399 (19)	0.92 (0.86-0.98)	1.06 (0.99-1.14)
Very high (>=1000)	458 (83)	95 (17)	0.78 (0.64-0.96)	0.98 (0.80-1.21)
Population-based quartiles				
Q1 (<19)	5,136 (77)	1,504 (23)	0.99 (0.93-1.06)	0.91 (0.85-0.97)
Q2 and 3 (19-128)	10,583 (79)	2,873 (21)	1.00 (Ref)	1.00 (Ref)
Q4 (>128)	5,134 (82)	1,155 (18)	0.86 (0.80-0.92)	1.02 (0.95-1.09)

*Adjusted for age (<30, 30-39, 40-49, 50-59, 60-69, 70+), sex (male, female), and education level (pre-secondary, secondary, post-secondary).

Fully adjusted analyses included 26,385 participants with complete covariate information.

Clinical categories reflect previously used IgE thresholds, whereas population-based categories were examined as a complementary exploratory analysis of the IgE distribution within AMORIS.

As an additional exploratory assessment of IgE across broader population distribution, IgE was also categorized using population-based quartiles. Individuals in the lowest quartile (<19 kU/L) had a modestly lower risk of cancer compared with the reference group (hazard ratio 0.91; 95% CI 0.85–0.97).

There was no evidence of violation of the proportional hazards assumption.

## Discussion

Our systematic review identified seven observational studies evaluating the association between ultra-low or deficient total serum IgE levels and cancer. Across these studies, most reported higher prevalence or risk of malignancy among individuals with IgE deficiency compared with non–IgE-deficient comparators, although the majority relied on retrospective or cross-sectional designs and reported largely unadjusted or minimally adjusted estimates. Study populations also varied substantially, ranging from general and tertiary-care populations to patients with primary immunodeficiencies. Only one study evaluated incident cancer risk using a longitudinal, multivariable, time-to-event framework and observed an increased risk of cancer among adults with IgE levels <3 kUI/L (21). In additional exploratory analyses using the AMORIS cohort, we found no evidence of increased overall cancer risk across clinically defined low IgE categories, although a modest inverse association was observed for the lowest population-based quartile. These findings should be interpreted cautiously given limited adjustment for potential confounding and the small number of individuals with ultra-low IgE. Taken together, the available evidence remains inconclusive, highlighting the need for larger prospective studies with comprehensive assessment of potential confounders to clarify whether IgE deficiency is independently associated with cancer risk.

Individuals with ultra-low or deficient total serum IgE constitute a heterogeneous group, and the clinical and immunological features underlying this phenotype remain incompletely characterized. IgE deficiency has been observed both in otherwise healthy individuals and in association with broader immune dysregulation (14, 24), including impaired humoral immune responses, recurrent infections, and, in some cases, primary immunodeficiency disorders (25). The recent USIDNET study further illustrates this complexity, reporting a higher prevalence of malignancy among IgE-deficient patients with CVID than among those without IgE deficiency (23). Very low IgE levels have been hypothesized to reflect altered immune surveillance, reduced antibody-mediated tumor recognition, or shared upstream immune regulatory pathways that could influence cancer susceptibility (2). However, it remains unclear whether IgE deficiency itself, or correlated aspects of immune function, underlie the associations reported in prior studies.

Taken together, several existing studies have reported associations in a similar direction between very low IgE levels and cancer occurrence across both pediatric and adult populations. Although this pattern should not be interpreted as consistent evidence of a well-established association, it provides an important basis for further investigation of ultra-low IgE and its potential relationship with cancer susceptibility. These observations raise the possibility that ultra-low IgE may reflect underlying immune alterations relevant to cancer susceptibility and could represent an important avenue for further investigation as a potential marker of immune dysregulation or cancer susceptibility (2, 12). However, consistency in the direction of findings should not be interpreted as consistency in the strength of evidence. The available literature is small and methodologically heterogeneous, with differences in study populations, definitions and thresholds used to classify IgE deficiency, comparator groups, timing of IgE measurement relative to cancer diagnosis, and adjustment for potential confounding. In particular, the use of different IgE thresholds (<2.0, <2.5, and <3 IU/L) may result in some individuals being classified differently across studies and therefore introduces potential exposure misclassification when findings are considered collectively. This variation also limits direct comparability of effect estimates and may contribute to differences in the magnitude of reported associations. Comparator definitions also varied, with some studies comparing IgE-deficient individuals with those having IgE levels within a specified normal range and others using broader non-deficient populations. These different reference populations further limit direct comparison of effect estimates across studies. Most studies were based on small numbers of individuals with IgE deficiency, relied on retrospective or cross-sectional designs, and primarily reported unadjusted comparisons of prior or prevalent malignancy (14, 18–20, 22, 23) rather than incident cancer occurring agter IgE measurement (21). In several studies, IgE was measured after cancer diagnosis, making the temporal relationship between IgE deficiency and malignancy difficult to establish and leaving open the possibility of reverse causation, including potential effects of malignancy or its treatment on subsequent IgE levels. Clinically selected study populations and differences in the indications for IgE testing also introduce the potential for referral or selection bias, while incomplete adjustment for underlying health and immune characteristics leaves the possibility of residual confounding. These considerations do not negate the observed associations but limit conclusions regarding their temporality, magnitude, and independence. Publication bias also cannot be excluded given the limited evidence base. Given the small number of studies and differences in their populations, designs, and reported IgE thresholds, meaningful sensitivity analyses stratified by IgE cut-off were not feasible. Therefore, the magnitude and independence of the association cannot be firmly established from existing studies. Further large, well-characterized prospective studies are needed to determine whether the observed association is reproducible and independent of underlying immune dysfunction and other potential confounding factors, and to determine whether associations differ according to the threshold used to define IgE deficiency.

We also observed differences in participant characteristics across IgE categories, including a higher proportion of women in the lower IgE groups. Although sex was included in the multivariable models, these differences underscore the importance of considering participant characteristics when interpreting associations between IgE and cancer risk. Several features of the AMORIS cohort may have limited its ability to detect associations involving ultra-low IgE, including a single baseline measurement of IgE, often decades prior to cancer diagnosis, limited availability of key covariates, and the absence of longitudinal or immunophenotypic data to characterize persistent IgE deficiency or broader immune dysfunction. Given the long follow-up period, changes in IgE concentrations over time may have resulted in exposure misclassification and potentially attenuated associations based on the baseline measurement. Adjustment was limited to age, sex, and education, and residual confounding by factors such as smoking, body mass index, allergic disease, immune dysfunction, and other health-related factors may have influenced the observed associations. In addition, IgE measurements were performed in the early 1990s, and although the assay permitted detection of very low IgE concentrations, differences in assay sensitivity and calibration relative to contemporary platforms may affect comparability across studies. Thus, the AMORIS findings provide additional exploratory evidence but should not be interpreted as demonstrating the absence of an association between IgE deficiency and cancer risk. Finally, despite the large overall cohort size, the number of individuals with ultra-low IgE was small, limiting statistical precision for this exposure group. Taken together, these findings highlight the need for large, well-characterized prospective cohorts with repeated IgE measurements and comprehensive assessment of potential confounding factors.

In addition to potential associations with cancer risk, growing evidence suggests that IgE may play a role in tumor immunosurveillance and could be leveraged therapeutically. These proposed mechanisms provide biological hypotheses that may help contextualize the epidemiological observations but are not established by the findings of the present systematic review. IgE antibodies are increasingly recognized for their potential role in cancer immunosurveillance and treatment, extending beyond associations with cancer risk alone. The immunosurveillance hypothesis proposes that IgE-mediated effector functions that operate against allergens may also function against tumor antigens or carcinogenic molecules, potentially contributing to the elimination of malignantly transformed cells before tumor development and indicating a potential role for IgE in anti-cancer responses (5, 26). While monoclonal antibodies of the IgG class are currently approved for use in cancer treatment, the potential immunosurveillance properties of IgE-based antibodies have drawn interest in the field of cancer immunotherapy research (2, 27, 28). IgE has several proposed theoretical advantages compared with IgG antibodies, including its ability to bind with high affinity to the Fcϵ receptor (FcϵRI), the lack of known inhibitory Fc receptors, and slower dissociation from Fcϵ receptors, resulting in longer retention on immune effector cells within tumors and potentially enhancing anti-tumor immune surveillance and cytotoxicity (29, 30). Preclinical studies exploring the use of IgE antibodies in cancer immunotherapies have shown promising results against antigens associated with melanoma, ovarian cancer, breast cancer, and prostate cancer (29, 31–33). More recently, a first-in-human phase I clinical trial of a first-in-class anti-cancer IgE (MOv18 IgE) reported encouraging findings in terms of safety and evidence of anti-tumor activity in ovarian cancer, although responses did not meet RECIST criteria (28). These experimental and early clinical findings are hypothesis-generating and do not establish therapeutic implications of the epidemiological associations examined in the present review. In addition to potential implications for cancer risk and treatment, emerging observational and translational evidence suggests that IgE-mediated immune mechanisms may also be relevant to tumor progression and survival, although findings remain limited and cancer-specific. Evaluation of prognosis-related outcomes was beyond the scope of the present study; however, future studies integrating longitudinal IgE measurements with cancer survival and treatment response data may help clarify the prognostic relevance of IgE biology.

This study has several strengths. To our knowledge, this is the first systematic review specifically focused on the association between ultra-low or deficient total serum IgE and cancer risk, providing a focused synthesis of an important but understudied area of research. The AMORIS cohort provides an additional exploratory evaluation in a large, population-based, prospective cohort with long-term follow-up and near-complete ascertainment of incident cancer through national registries, minimizing outcome misclassification. All serum IgE measurements were performed at a single central laboratory using a standardized and consistent methodology, reducing inter-laboratory variability. Importantly, the assay had a very low lower limit of detection, allowing identification of individuals with ultra-low IgE levels, an exposure that cannot be reliably captured in many epidemiologic cohorts. However, several limitations should also be considered. In the systematic review, the search was limited to English-language publications and did not systematically search grey literature, dissertations, or trial registries, which may have introduced language and publication bias. The review was not prospectively registered and did not have a pre-specified protocol. The small number and heterogeneity of included studies, including differences in study populations, definitions of IgE deficiency, comparator groups, study design, and analytic approaches, precluded quantitative meta-analysis and formal assessment of publication bias. In addition, most studies evaluated prior or prevalent malignancy rather than incident cancer occurring after IgE measurement, limiting assessment of temporality and increasing the potential for reverse causation. Findings were therefore interpreted narratively in the context of study design, confounding adjustment, and risk of bias, and a formal certainty-of-evidence assessment was not performed. Taken together, these factors constrain causal inference, and findings should be interpreted with appropriate caution. The exploratory AMORIS analyses should also be considered in light of several important limitations. Serum IgE was measured only once at baseline, often decades prior to cancer diagnosis. Given the median follow-up of approximately 23 years, changes in IgE levels over time could have resulted in exposure misclassification and attenuation of associations, and a single measurement could not establish persistent IgE deficiency. In addition, the underlying clinical or occupational reasons for IgE testing were not available in AMORIS. Individuals undergoing IgE testing may differ systematically from the broader population because of allergic disease, chronic inflammatory conditions, immune dysfunction, healthcare utilization, or physician referral patterns, introducing potential indication or selection bias. Consequently, associations observed within the AMORIS analytic sample may not necessarily reflect those that would be observed in an unselected general population. Although the AMORIS assay permitted detection of very low IgE concentrations, variation in IgE assay platforms, calibration, and limits of detection represents an inherent source of measurement heterogeneity that may attenuate observed associations across studies. Moreover, the historical Swedish setting of AMORIS may limit the generalizability. Participants were sampled between 1985 and 1996, and changes in cancer epidemiology, diagnostic practices, and healthcare delivery may affect applicability to contemporary populations. The Swedish population and healthcare context may also differ from more demographically diverse populations and healthcare systems. In addition, differences in IgE assay platforms, calibration, and limits of detection may affect comparability across settings and time periods. Multivariable adjustment was limited to age, sex, and education, and incomplete information on important potential confounders, including body mass index, smoking status, and other lifestyle and clinical factors, raises the possibility of substantial residual confounding. Because these factors are associated with cancer risk and may also be related to IgE levels, residual confounding may have influenced the observed null associations. The analysis focused on overall cancer risk, and evaluation of associations by specific cancer type was limited by small numbers of cases among individuals with ultra-low IgE, despite the large cohort size. Finally, the study relied on total serum IgE alone, without data on other immunoglobulin classes or clinical allergy and immunodeficiency phenotypes, limiting the ability to distinguish isolated IgE deficiency from broader immune dysfunction. Taken together, these limitations constrain causal inference, and the AMORIS findings should be considered exploratory rather than definitive evidence regarding the association between IgE deficiency and cancer risk.

In conclusion, this systematic review synthesizes the limited evidence in an understudied area of potential clinical and immunological relevance. Several published studies have reported higher cancer prevalence or risk among individuals with ultra-low or deficient total serum IgE, although the existing evidence is limited by small numbers, heterogeneity across studies, and largely unadjusted analyses. In additional exploratory analyses using the AMORIS cohort, we found no evidence of increased overall cancer risk among individuals with clinically defined IgE deficiency; however, these findings should be interpreted in light of the limitations of the available data. Taken together, these findings suggest that the relationship between IgE deficiency and cancer risk remains unresolved. Future large, population-based prospective studies with repeated IgE measurements and comprehensive clinical and lifestyle covariate data will be important to better clarify this association and determine whether IgE deficiency may have relevance for cancer risk assessment.

## Data Availability

Data from the AMORIS cohort are not publicly available due to data protection restrictions. Access may be granted upon application and approval by the relevant authorities. Requests to access these datasets should be directed to https://ki.se/imm/forskning-vid-imm/enheter-vid-imm/enheten-for-epidemiologi/for-researchers.
